# Automatic characterization of stride parameters in canines with a single wearable inertial sensor

**DOI:** 10.1371/journal.pone.0198893

**Published:** 2018-06-14

**Authors:** Gregory J. Jenkins, Chady H. Hakim, N. Nora Yang, Gang Yao, Dongsheng Duan

**Affiliations:** 1 Department of Molecular Microbiology and Immunology, School of Medicine, University of Missouri, Columbia, Missouri, United States of America; 2 Department of Bioengineering, University of Missouri, Columbia, Missouri, United States of America; 3 National Center for Advancing Translational Sciences, Bethesda, Maryland, United States of America; 4 Department of Veterinary Biomedical Sciences, College of Veterinary Medicine, University of Missouri, Columbia, Missouri, United States of America; 5 Department of Neurology, School of Medicine, University of Missouri, Columbia, Missouri, United States of America; University of Illinois at Urbana-Champaign, UNITED STATES

## Abstract

**Background and objective:**

Gait analysis is valuable for studying neuromuscular and skeletal diseases. Wearable motion sensors or inertial measurement units (IMUs) have become common for human gait analysis. Canines are important large animal models for translational research of human diseases. Our objective is to develop a method for accurate and reliable determination of the timing of each stride in dogs using a wearable IMU.

**Methods:**

We built a wireless IMU sensor using off-the-shelf components. We also developed a MATLAB algorithm for data acquisition and stride timing determination. Stride parameters from 1,259 steps of three adult mixed breed dogs were determined across a range of six height-normalized speeds using the IMU system. The IMU results were validated by frame-by-frame manual counting of high-speed video recordings.

**Results:**

Comparing IMU derived results with video revealed that the mean error ± standard deviation for stride, stance, and swing duration was 0.001 ± 0.025, -0.001 ± 0.030, and 0.001 ± 0.019 s respectively. A mean error ± standard deviation of 0.000 ± 0.020 and -0.008 ± 0.027 s was obtained for determining toe-off and toe-touch events respectively. Only one step was missed by the algorithm in the video dataset of 1,259 steps.

**Conclusion:**

We have developed and validated an IMU method for automatic canine gait analysis. Our method can be used for studying neuromuscular diseases in veterinary clinics and in translational research.

## Introduction

Gait analysis is valuable for diagnosis and assessing disease progression/therapy response in diseases that alter gait, such as Duchenne muscular dystrophy [1]. Observer-based examination has conventionally been used in gait assessment and is standard in clinic diagnosis [2]. However, subjective approaches are difficult to generate precise measurements. Quantitative methods such as optical recording systems, ground force plates/pressure walkways, and electromyography have become more popular in gait analysis [3]. However, these technologies require expensive and sophisticated instrumentation and facilities which limit their access for routine application. Low-end motion capture systems, such as OptiTrack (NaturalPoint, OR, USA), may cost ~$15,000 USD; while high-end video systems such as the Vicon system (Vicon, Oxford, UK) may run more than $200,000 USD [4]. Pressure walkways such as the GAIT4Dog system (CIR Systems, NJ, USA) may cost ~$25,000 USD.

Recently, wearable inertial sensors or inertial measurement units (IMUs) have gained attention in motion analysis for their small size, low cost (usually < $500 USD), and capability to reveal 3D motion. IMUs typically contain accelerometers, gyroscopes, and magnetometers conventionally used in navigation systems. IMUs are becoming well-established technology for human gait studies [5]. Several groups have explored wearable IMUs in canine, a critical species to study human diseases in large animals [6].

The majority of canine IMU gait studies has relied on a single device attached dorsally or ventrally to the chest, back, or neck of the dog to measure bouncing and swaying of the body during motion [7, 8, 9, 10, 11]. Alternatively, several studies have analyzed canine gait by one or more limb-mounted IMUs [12, 13, 14, 15]. Limb-mounted techniques directly quantify metrics such as step phase durations, joint motions, and gait type.

Gait can be described as alternating phases of swing (time the paw is in the air) and stance (time the paw is on the ground). A complete movement through swing and stance phase is a stride or one gait cycle. A core issue in gait analysis is to accurately and reliably define the phases of the gait cycle, as this sets the foundation for gait type characterization and stride parameter quantification.

Of the limb-mounted IMU studies in dogs, only two groups have proposed strategies to measure timing of step phases. Wilshin et al. used the change in rotational velocity detected by a gyroscope to indicate the start and end of swing phase [13]. However, the accuracy of the method was not validated. Alternatively, Ladha et al. used acceleration and velocity from an accelerometer and compared the IMU results with video recording to determine accuracy [14]. However, their system left room for improvement due to frequent step misidentification and significant timing error in the evaluated steps.

In this study, we discovered new signal markers to reliably and accurately identify step phase timing of the canine forelimb via IMU. A video-recorded treadmill study validated our algorithm at multiple speeds. The method we developed represents a significant improvement over the published methods.

## Materials and methods

### Wearable wireless sensor

**Fig 1:**
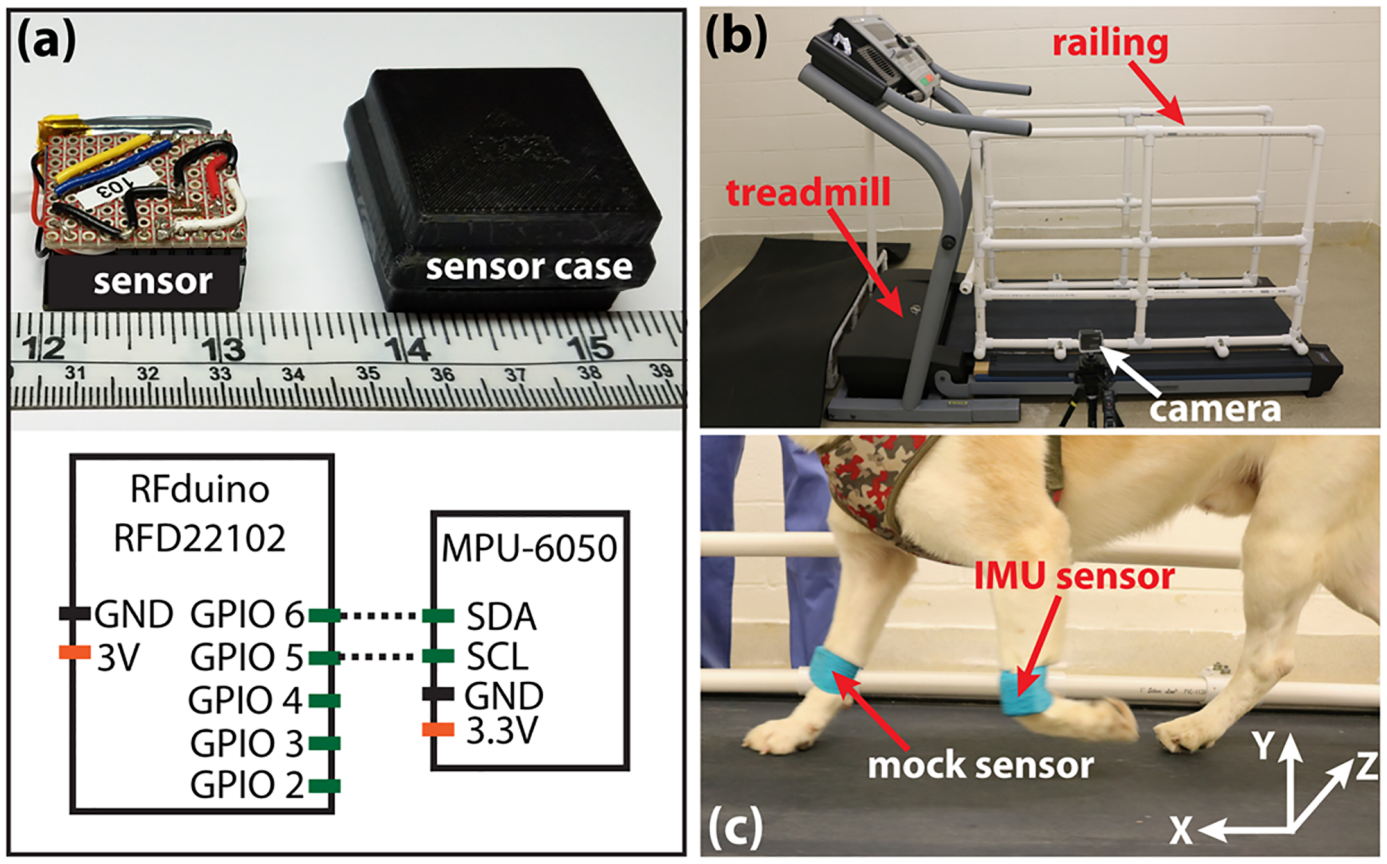
An overview of the IMU-based dog gait analysis system. (a) A photo of the IMU sensor and the container and a schematic of the sensor circuit. (b) A photo of the experimental setting. (c) A photo illustrating sensor position and axes orientation.

The gait analysis sensor was assembled in-house using a six-degree accelerometer and gyroscope sensor board (MPU-6050, InvenSense, San Jose, CA, USA), a Bluetooth enabled microcontroller (RFD22102, RF Digital Inc., Hermosa Beach, CA, USA), and a 3.7 V 100 mAh battery. The accelerometer detected tri-axial acceleration within a range of ±16 g (1 g = 9.81 m/s^2^), and the gyroscope detected tri-axial angular velocity within a range of ±2000 °/sec. The sensor was housed in a custom-designed 3D-printed plastic case. The entire package had a weight of 20 grams, and a size of 20 mm by 35 mm by 35 mm (Fig 1). Data was streamed from all six inertial axes simultaneously at 100 Hz via the Bluetooth transceiver to an external laptop computer. The microcontroller was programmed using open-access Arduino software. Programs to operate the sensor and interpret the data were developed using MATLAB software (The MathWorks Inc., Natick, MA, USA).

### Animals

All animal experiments were approved by the Animal Care and Use Committee of the University of Missouri and were performed in accordance with National Institutes of Health guidelines. Three dogs were used in the study to test the sensor and algorithm. All were studied at the age of 6 months. Two dogs were studied again at the age of 9 months. The average height at withers (HW) was 42 ± 2.5 cm at 6 months and 44.5 ± 3.6 cm at 9 months. The average weight was 17 ± 3 kg at 6 months and 21 ± 4 kg at 9 months. Dogs used in the study were of a mixed genetic background of golden retriever, Labrador retriever, beagle, and Welsh corgi and were generated in house by artificial insemination.

### Treadmill protocol

A NordicTrack c2200 treadmill (NordicTrack, Inc., Logan, UT, USA) was used in this study. To aid in dog cooperation and safety, a polyvinyl chloride railing was installed on both sides of the treadmill and an exit ramp was added to the front (Fig 1).

On the day of a gait test, the height at withers (HW) was measured from the ridge between the shoulder blades (the withers) to the ground. This measurement was used to calculate height-normalized speed settings for the treadmill, defined as speed (m/s) / height (m). The HW-normalized speed values of 1.5, 2.0, 2.5, 3.0, 3.5, and 4.0 s^-1^ were used in this study. For example, a 0.5 m HW dog would need the treadmill set to 2.0 m/s to move at a HW-normalized speed of 4.0 s^-1^. Such a normalization process was used to reduce the effect of height on gait parameters [8].

The IMU was mounted directly above the carpal joint on the lateral side of the left forelimb using a 12-inch strip of cohesive bandage (VetWrap, 3M, Maplewood, MN, USA) (Fig 1). The device was always oriented with the positive x-axis pointed forward (toward the anterior side of the dog), the positive y-axis pointed upward (toward the dorsal side of the dog), and the positive z-axis pointed towards the midline (medial side) of the dog. A mock sensor was similarly attached to the right forelimb to equalize weight distribution (Fig 1).

Following sensor mounting, the dog was allowed a few minutes to acclimatize to wearing the device. Once comfortable, the dog was asked to mount the treadmill. The IMU started recording and the treadmill was started at the lowest HW-normalized speed. After 60 seconds, the treadmill and sensor were stopped, and the dog dismounted the treadmill for 3–5 minutes of rest. This process was repeated for each of the six HW-normalized speeds from the lowest to the highest (1.5 to 4.0 s^-1^).

The movement of the dog was recorded using a 120 fps video camera (GoPro Hero 3+ Silver Edition, GoPro, Inc., San Mateo, CA, USA) positioned near the side of the treadmill (Fig 1). The sensor and camera recordings were synchronized by shaking the sensor-equipped limb three times at the beginning of each test.

### Video analysis

Video recordings were reviewed manually frame-by-frame to determine the timing of toe-off and toe-touch events for every stride. Toe-off was marked when the paw clearly lost contact with the ground. Toe-touch was marked when any part of the paw regained contact with the ground. The duration for each swing, stance, and stride was calculated by converting number of frames to seconds.

About 50–60 consecutive strides were counted for every dog at every speed obtained at every age time point. A total of 1,306 steps were counted, of which 47 steps were removed from analysis due to gait-affecting external interferences. Steps were excluded when hindlimbs fell off the back of the treadmill (21 steps), the dog received a treat (15 steps), video footage was obscured by the railing (8 steps), or the leash was pulled (3 steps). Of the remaining 1,259 accepted strides, 180 steps were associated with behavioral irregularities, but were kept in the analysis. These activities included the dog looking around or sniffing the ground (113 steps), jumping (26 steps), momentarily stopping (12 steps), or other irregularities (29 steps). The steps with regular gait type were labeled as either walk (739 steps) or trot (340 steps). Walk gait type included strides with predominantly three limbs on the ground at a time. Trot gait type included strides with diagonally paired limbs in identical movements.

### Automatic step detection

Each gait test file contained about 60 s of sensor data sampled simultaneously at 100 Hz in each of the six IMU axes. The first and last five seconds of recorded IMU data were removed from the comparison because the treadmill was speeding up or slowing down during these periods. The raw signals from all axes were first interpolated to 1,000 Hz using cubic spline interpolation before further processing. The 1^st^ order derivative of the signal was then calculated after the signals were filtered using a 3^rd^ order Savitzky-Golay filter with a frame size of 0.051 s.

**Fig 2:**
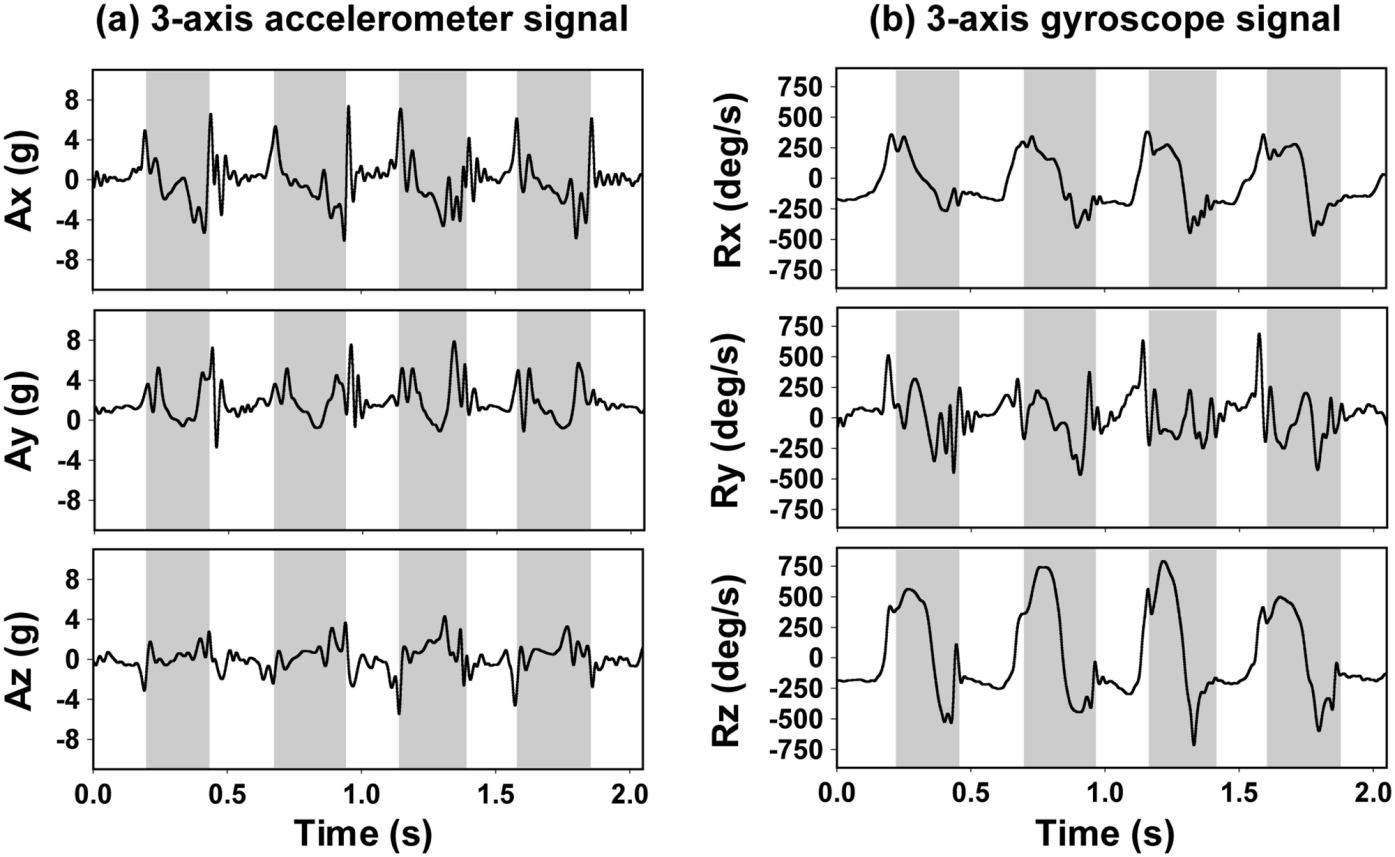
Representative tracing from the sensor. (a) Accelerometer readings. Signals are expressed as the g-force (9.81 m/s^2^). (b) Gyroscope readings. Signals are expressed as the angular velocity (deg/s). Shaded regions indicate swing phase of the limb.

**Fig 3:**
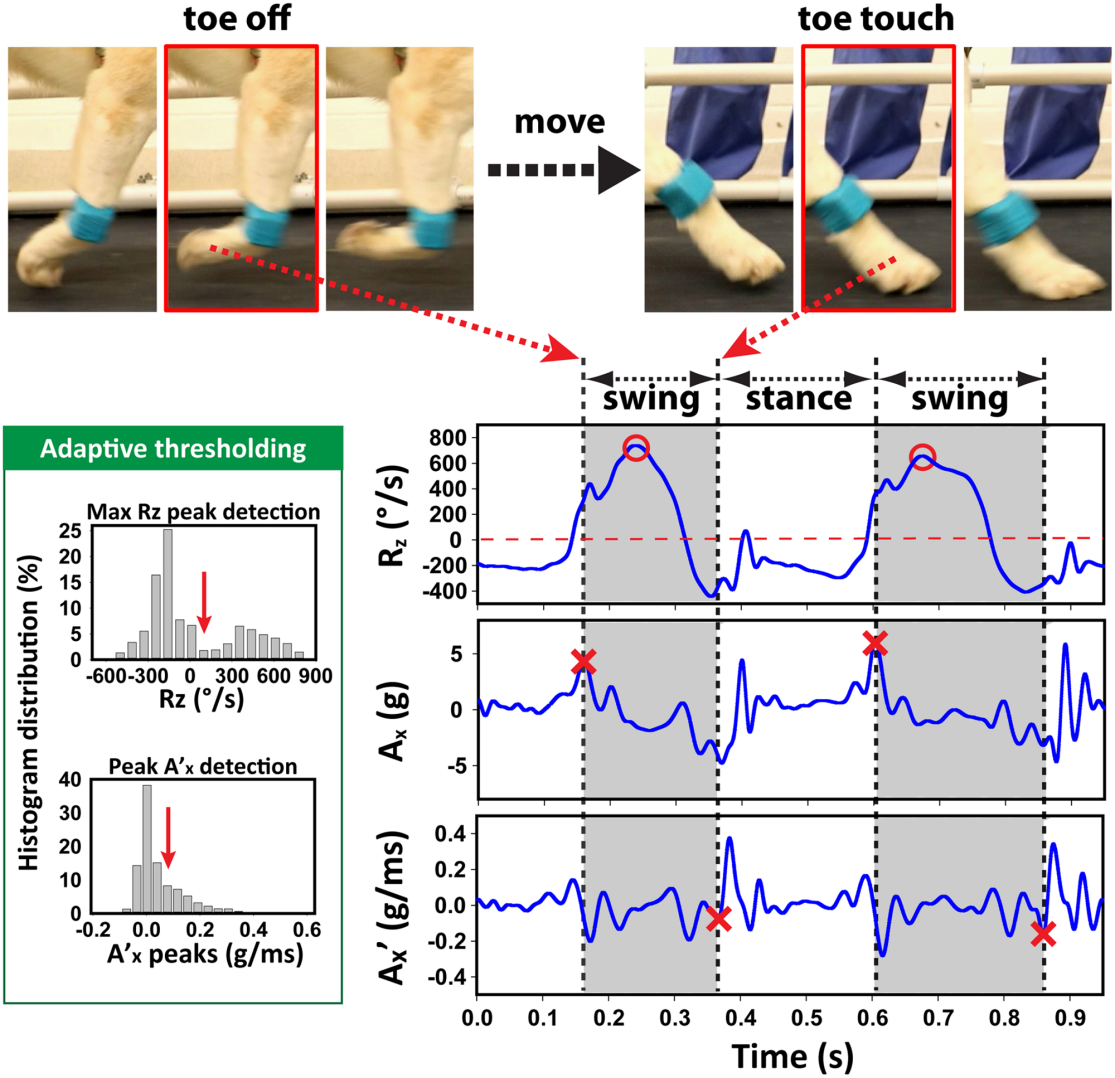
Method for detecting swing start and end. (Top) Frame-by frame real-life images from video recording (images were mirrored so that the frame sequence matches the direction of motion and time). (Bottom right) Representative tracing of the z-axis angular velocity (Rz), x-axis acceleration (Ax) and the first derivative of Ax (Ax’). Circles indicate the Rz peak. The “cross” symbols indicate the start of swing and stance, respectively. (Bottom left) Charts depicting the histogram distribution of peaks in Rz and Ax’ for setting detection thresholds away from the signal baseline.

Gait cycle related motion patterns were noticed in all six axes (Fig 2). Among these, the z-axis angular velocity (Rz) showed a strong and consistent profile with every step and was used as the signal marker to identify a step (Fig 2b). The Rz signal represented the primary rotating motion of the forelimb and appeared as a broad and dramatic peak (swinging forward) followed by a smaller negative profile (touching the ground). To detect a step, the “findpeaks” function in our custom-programmed MATLAB algorithm was applied to identify maximum peaks in Rz using a baseline threshold and a minimal separation of 0.3 s. The identified peaks were marked and used as a localizer for finding the start of swing and stance phases (Fig 3).

Toe-off (the instant the paw leaves the ground and initiates swing phase) coincided with a peak in the x-axis acceleration signal (Ax). Therefore, a sharp maximum in the Ax signal (just before peak Rz) was used to define the start of swing phase. This feature was highly consistent and easy to obtain for all steps.

Toe-touch (the instant the paw touches the ground and initiates stance phase) occurred immediately before a major peak in Ax’ (the first derivative of Ax). The toe-touch event was more complicated to pinpoint. To begin, the algorithm looks ahead from the identified peak in Rz for the time point when the Rz signal crosses the zero rotational speed. The sign change in Rz indicates a change in rotation of the limb from forward to backward. From the zero-crossing time point, the algorithm looks ahead to find the time point when Rz reaches its minimum. Next, the algorithm finds the latest peak in Ax between the zero-crossing and the minimum Rz. The algorithm then looks ahead from the peak in Ax to find the first major peak in Ax’. The peak in Ax’ must be greater than a threshold based on the signal baseline; if none are accepted, the largest peak will be used. The algorithm then moves to the minimum in Ax’ immediately before the aforementioned peak Ax’ to mark the time point of toe-touch.

The thresholds for detecting peak Rz and peak Ax’ were determined independently for each gait file by analyzing the histogram distribution of the signals (Fig 3, bottom left). The Rz histogram exhibited a bimodal distribution due to the stance phase taking on negative values and the swing phase corresponding to large amplitude positive values. Therefore, the Rz peak threshold (for step detection) was set at trough between the two clusters in the Rz histogram so that all negative peaks as well as the baseline signals were excluded in Rz peak detection. The Ax’ histogram was peaked near zero and gradually decreased towards larger positive values. The Ax’ threshold, for detecting swing end, was empirically set at two histogram bins following the peak distribution in the histogram of all Ax’ peaks. Our results suggested that this threshold was effective in preventing an insignificant peak from being labeled incorrectly as a swing end event.

The algorithm also defined a search window to search for the toe-off and toe-touch events. The search window was based on the stride frequency, the location of the following step peak Rz, and the location of the previous step toe-touch. The search windows limited the maximum potential for error and decreased the chances of a preceding or following step event from interfering with the algorithm identifying the current step. To search for swing start, the search window is set to one third of the average stride duration before the current peak in Rz. To search for stance start (or swing end), the search window is set to two thirds of the distance towards the subsequent step’s peak in Rz. If a swing start was placed within 0.15 s of a prior stance start, this would likely indicate an error; and the search window for swing start would be reduced in size by half and swing start would be relocated. For the last step or if no steps were taken for greater than 2 s, one half the average stride duration was used to define the swing end search window after the current peak Rz.

**Fig 4:**
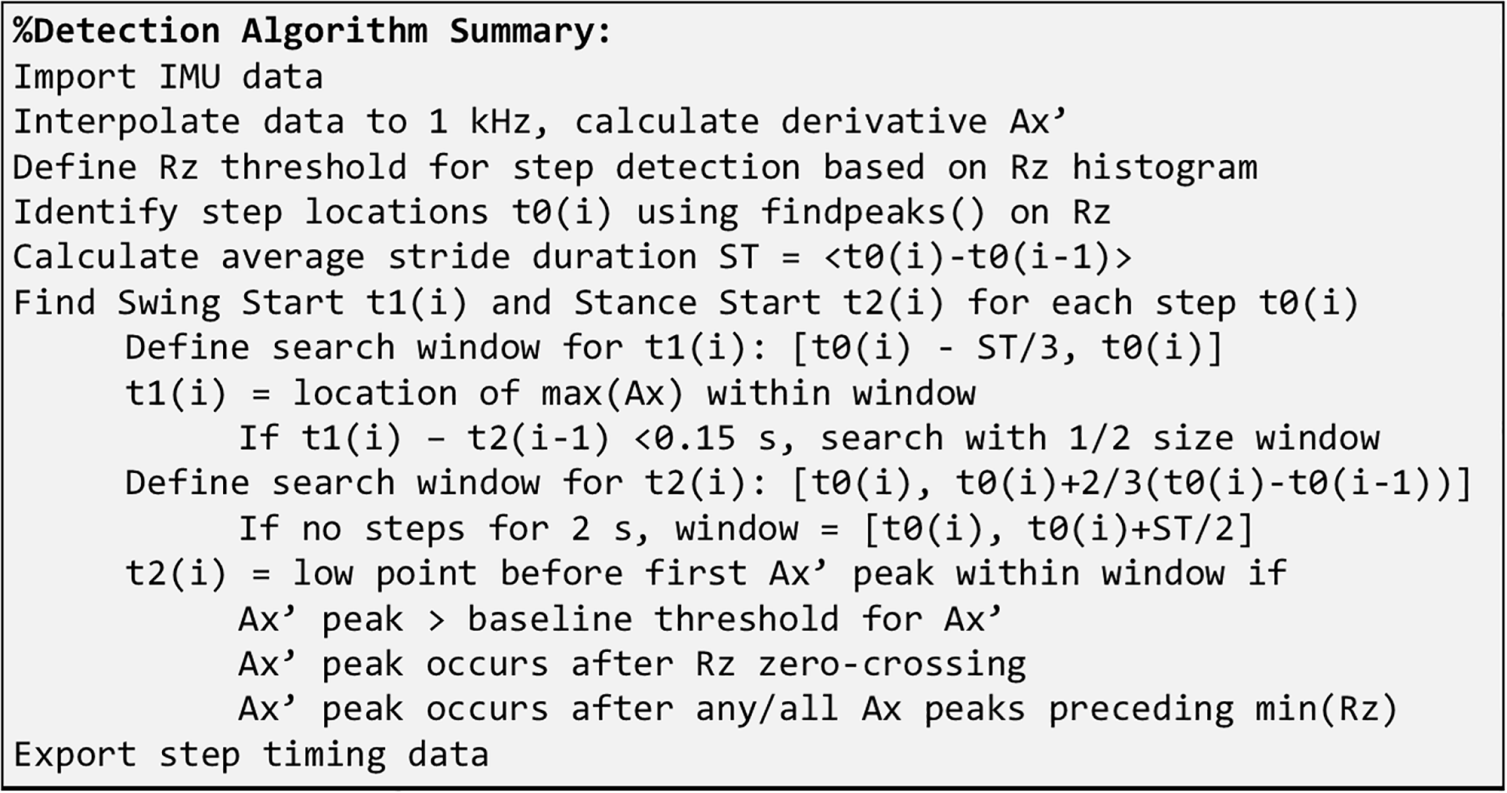
A summary illustration of the step detection algorithm.

The algorithm described above was implemented in MATLAB software (The MathWorks Inc., Natick, MA, USA). The major steps used in the algorithm were summarized in Fig 4.

### Validation

The video and IMU datasets were synchronized for data analysis by matching the video paw shake to the corresponding event in the Rz signal at the start of every recording. To evaluate the accuracy of the algorithm to detect the events of toe-off and toe-touch, the first identified swing start in the video and in the sensor data was set as their corresponding time zero. The time points of subsequent toe-off and toe-touch events were compared step-by-step between the video analysis and sensor analysis. The detection error was calculated by subtracting the corresponding manually counted video result from the automatically detected result. In addition, the accuracy of the sensor-defined stride, stance, and swing durations was also analyzed by comparing step-by-step with the video results. For each step, the swing duration was calculated as the time difference between the toe-off and the subsequent toe-touch; the stance duration was calculated as the time difference between the toe-touch and the subsequent toe-off; and the stride duration was calculated as the time difference between two consecutive toe-off events. S1 Dataset contains the step counting results.

## Results

To develop an automatic method that can accurately detect the swing and stance phase of a gait, we evaluated signals from the sensor. Characteristic patterns were observed in all axes from both accelerometer and gyroscope (Fig 2). To guide programming, a subset of video data was used to correlate the signal pattern and real-life dog gait. A broad Rz peak was consistently observed in every step. Hence, this was used as the step identifier. To detect swing and stance start, we explored a series of signal features from every axis. The preliminary algorithm was then tested in the whole video collection for the best fit. Feedback was then used to optimize the algorithm. Through iterative cycles of data fitting, we identified the best signal markers for detecting the swing and stance start (Fig 3).

**Fig 5:**
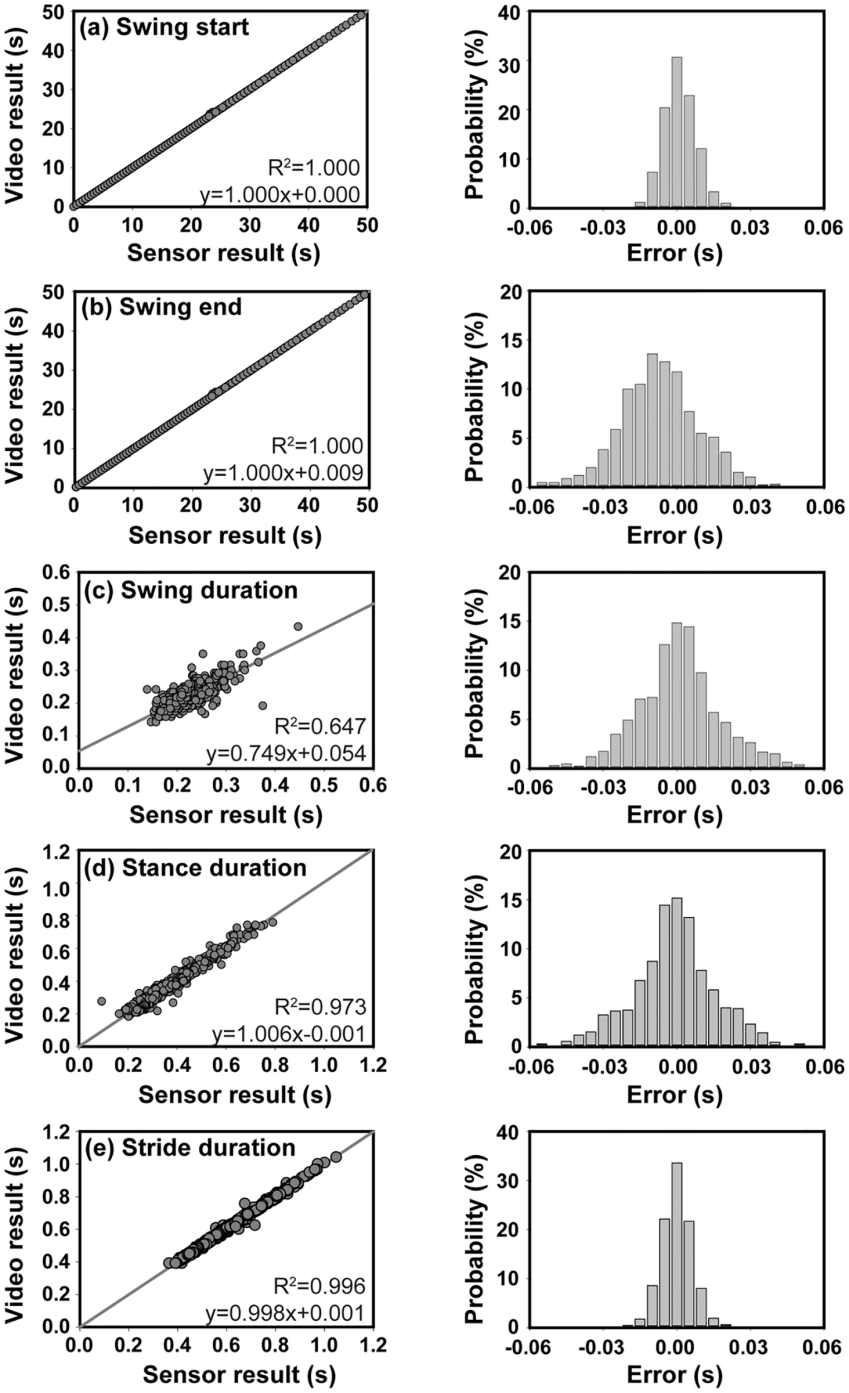
Quantitative comparison of video results and sensor results for (a) swing start (toe-off event), (b) swing end (toe-touch event), (c) swing duration, (d) stance duration, and (e) stride duration. Left and right panels depict the linear correlation and step-by-step error distribution, respectively, between the video and sensor results.

A quantitative comparison of the toe-off, toe-touch, swing duration, stance duration, and stride duration between the automatic sensor detection and manual video analysis revealed a strong performance of the algorithm (Fig 5). For each parameter, the linear correlation and distribution of the step-by-step errors between the video and sensor results were analyzed. A total of 1,259 strides were used in the comparison. The coefficient of determination (r^2^) reached 1.000 and 1.000 for the detection of toe-off and toe-touch events, respectively. Analysis revealed a mean error ± standard deviation of 0.000 ± 0.020 and -0.008 ± 0.027 s for determining toe-off and toe-touch events respectively.

The coefficient of determination (r^2^) reached 0.996, 0.973 and 0.647 for the stride, stance and swing durations, respectively. The lower r^2^ for the swing duration was mainly attributed to the significantly smaller data range. For perspective, the average duration for the stride, stance, and swing was 0.575, 0.355 and 0.221 s. The mean error ± standard deviation between the video and sensor results was 0.001 ± 0.025, -0.001 ± 0.030, and 0.001 ± 0.019s for the stride, stance and swing duration, respectively. Only one instance of a step being counted in the video but missed by the algorithm was observed. In this particular case, the prior step appeared to have an extra-long stance phase.

**Fig 6:**
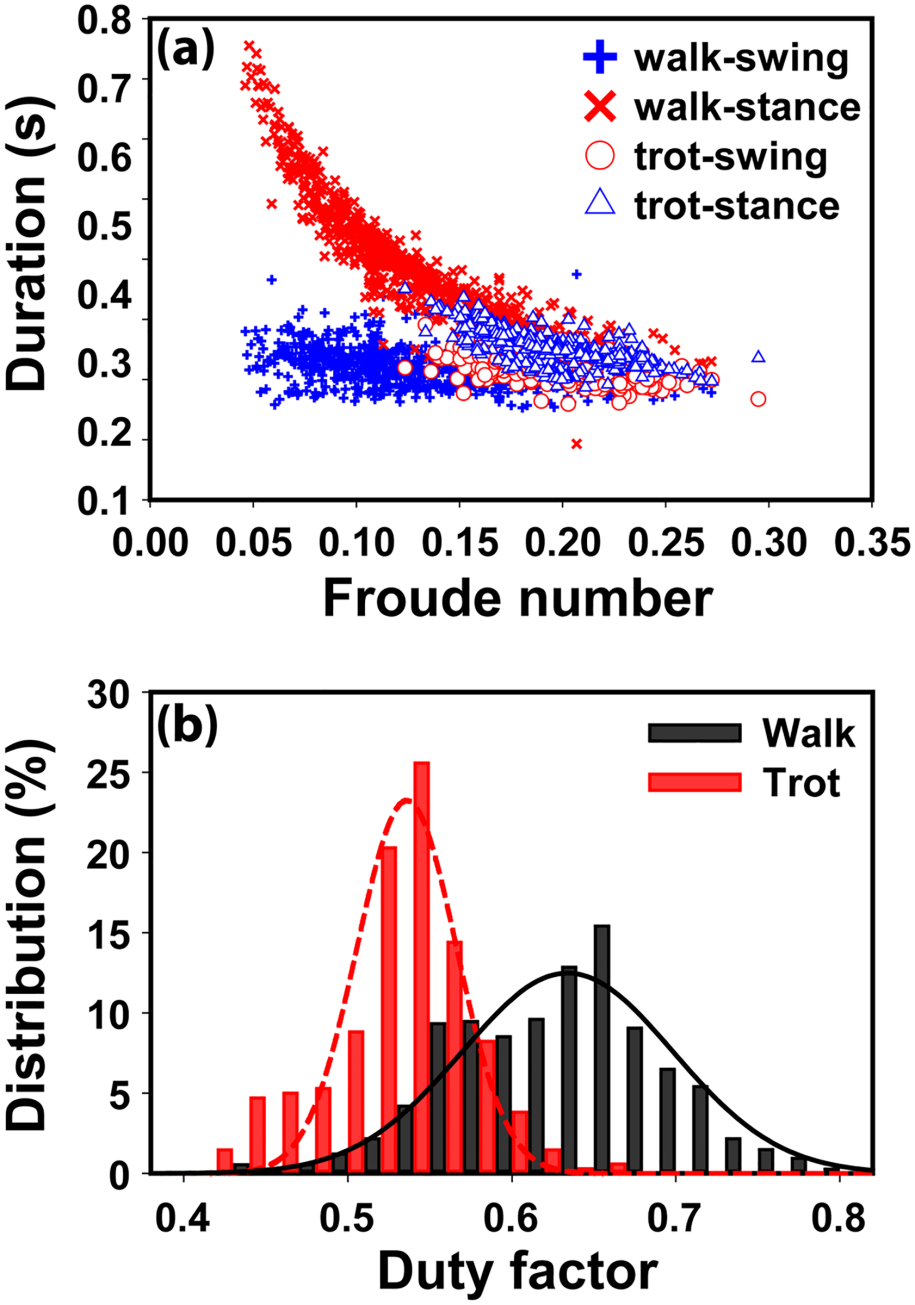
The step phase duration as a function of Froude number and the distribution of duty factor in walk and trot gait types. (a) The stance and swing durations at different Froude numbers. (b) The distribution of the duty factor for trot and walk gait types. The gait type (walk or trot) was identified in video analysis. The lines are best Gaussian fit: y = 0.23exp[-544.61(x-0.54)^2^], R^2^ = 0.98 for trot gait, and y = 0.12exp[-120.19(x-0.63)^2^], R^2^ = 0.94 for walk gait.

To determine whether our method can yield similar results to those obtained using a field-standard technique, we analyzed the relationship between Froude number, stride phase duration (stance and swing), and gait type (walk or trot) (Fig 6a). The “Froude number” is a dimensionless number calculated as h*f ^*2*^*/g*, where *h* is the height at withers, *f* is the stride frequency, and *g* is gravity [16]. It is broadly used in gait analysis to allow a normalized comparison between different studies [17]. By plotting the step phase durations against the Froude number for each stride, we obtained a nearly constant value for swing duration irrespective of the Froude number, while the stance duration became shorter with the increase of the Froude number. The trajectories obtained for swing and stance duration and the occurrence of trot gait type at their intersection agreed very well with findings obtained by others using a manual optical recording technique [18].

We also examined the relationship between duty factor and gait type (Fig 6b). For gait analysis, the duty factor is defined as the percentage of the stance duration of the entire stride duration. We found that the distribution of the duty factor can be well fitted using a Gaussian distribution in both walk gait (R^2^ = 0.94) and trot gait (R^2^ = 0.95). The trot gait duty factor ranged predominantly from duty factors of 40–60%, while the walk gait occurred mainly at duty factors of 50–80%. Similar results had been described in the dog gait study literature using the field-standard pressure sensing walkway [19].

## Discussion

Gait analysis systems are beneficial to researchers of neuromuscular and skeletal diseases, and the canine is an important model for translational research of human diseases [6, 20]. State-of-the-art methods such as pressure walkways and video motion-capture are highly capable of measuring canine gait [21]; yet, wearable IMUs offer distinct advantages due to their unique capabilities [22]. Specifically, pressure walkways are disadvantaged in that they only obtain gait characteristics at the 2D surface of the walkway; whereas IMUs obtain complete 3D motion even while the leg is off the ground. Video motion-capture is disadvantaged because it can only obtain a few strides at a time (unless coupled with a treadmill) and it requires a time-consuming setup to precisely attach anatomical markers. Additionally, video methods often obtain side-view 2D analysis only. A true 3D analysis requires a dedicated testing facility and complex multi-camera setups. Considering the low-cost, simplicity, capability, and added benefit of wearable IMUs to function in any environment, a sufficient IMU system would be able to achieve the same functionality in all existing gait analysis systems.

In this study, we developed a method to automatically measure stride phase times. Stride phase timing is a key parameter in gait analysis [23]. Our method obtained phase timing substantially better than IMU methods published by other groups. Wilshin et al. used a sign change in the rotational velocity of limb-mounted sensors to mark gait phase changes in canines [13]. Although the authors did not validate their method, our analysis clearly showed that the author’s method would produce large error in determining swing and stance duration. For comparison, we re-analyzed our data with their criteria and the results revealed an underestimation of the swing duration with a mean error of >40 ms (S1 Fig). Ladha et al. used a peak in the forward acceleration and a minimum in forward velocity to define the start and end of the swing, respectively [14]. Ladha et al. also used video to test accuracy and reported a mean error of ~38.5 ms for forelimb toe-off detection in walking dogs. As a comparison, our mean error was 0.000 ms with a 10 times smaller error distribution. Another aspect is the ability to correctly identify a step. Ladha et al. reported that their algorithm correctly detected a step 83% of the time. With our algorithm, we were able to correctly detect a step 99.92% of the time (only missed one video step and made zero false steps in the 1,259 counted steps). We did not attempt to re-analyze our data with the Ladha et al. criteria, as their swing phase end detection relied upon a drift-prone integration step.

The gait profile may be affected by the mounting position and orientation of the sensor. Rhodin et al. attached the sensor on the metacarpal bones [15]. It was shown that inconsistent marker placement for video motion-capture systems contributed the greatest source of variability in human gait test centers [24]. We found it difficult to mount the device anywhere below the carpus because it obstructed walking. Hence, we mounted the sensor on the forearm above the carpal joint. The surface of the forearm is slightly curved, and obtaining a consistent device orientation was difficult. Although minor mounting inconsistency was inevitable among animals, they did not significantly affect all major signal features described in this study. However, exploration of mounting methods that mimic the musculoskeletal shape of the forearm (such as a moldable strap) should improve the mounting consistency and may further improve our method.

The event of toe-touch was difficult to obtain consistently. Difficulty arose from frequent jerkiness in the signal around the moment of impact with the ground that could generate false peaks for the detection algorithm. The rationale behind the algorithm for detecting toe-touch was to get as close as possible to the toe-touch indicating peak in Ax’ without passing the event. The regime where Rz has crossed the zero axis and is becoming increasingly negative must be before the toe-touch impact influences the device. Therefore, we used the first major peak in Ax’ after this regime as the toe-touch indicating peak. Further difficulty arose from the fact that the force imparted by the ground during toe-touch must transfer along the paw and joints before influencing the wrist mounted sensor. Nonetheless, our algorithm obtained toe-touch with high consistency and accuracy when compared with the video results.

Considering our study was conducted on a treadmill, the result may exhibit more consistent step identification due to the consistent speed of the gait. As commonly practiced in the canine studies [25], we trained the dogs used in the study to ensure they were habituated with treadmill locomotion. Our method was only validated in dogs of similar sizes. However, we included a normalization step in treadmill speed setting to accommodate for the difference in the dog height. In addition, all detection thresholds used in our method were automatically adjusted based on each individual gait data profile. Previous results [8, 19] also suggest that dogs of different sizes showed similar gait features after normalizing the speed against height. Collectively, we believe our method should not be affected by any size-related signal amplitude changes. Nevertheless, tests in more dogs with bigger variations in size will be helpful to experimentally validate the performance consistency of our method. A future study should also be conducted to analyze system performance with over-ground locomotion. Additionally, this study can be extended to examine all four limbs that will allow comprehensive analysis of canine gait.

In real world or free-roam environments, external influences and unexpected events will occur during the data collection. In this study, 47 steps were removed due to perceived external factors such as treating the dog or pulling on the leash. Yet, the performance of our algorithm was still very strong for these 47 steps. The accuracy for swing and stance event detection for these 47 steps alone was 0.002 ± 0.009 and 0.012 ± 0.022 s respectively. By including these steps in the overall analysis, the result for swing and stance event detection becomes 0.000 ± 0.020 and 0.002 ± 0.027 s respectively. Nevertheless, the accuracy of the stride parameter detection may be best maintained in practical applications by profiling all detected steps so that only steps from regions of steady gait with a consistent stride frequency are included in the analysis.

## Conclusion

A wearable IMU method with sufficient capability could be preferable to state-of-the-art methods due to the low-cost and convenience of IMUs. We have developed an automated step phase timing detection method for wearable IMU-based dog gait analysis. Our IMU system offers high precision at 100 Hz data acquisition and obtained stride phase timing within a very small margin of error. To demonstrate whether our results were consistent with findings from field-standard techniques, we evaluated the correlation between step duration and the Froude number as well as the distribution of the duty factor over gait type. We found our results were remarkably similar to those that used pressure walkway or video recording techniques. Further development of our method is warranted for future application in veterinary clinics and in preclinical studies of neuromuscular diseases using the canine model.

## Supporting information

S1 Dataset Video frame raw counting data and MATLAB export. The anonymized dataset of all video counted steps and their corresponding MATLAB predictions are contained in the file “S1_Dataset.xlsx”.
(XLSX)

S1 Fig Results from re-analyzing data by Wilshin et al. criteria. (a) Step-by-step error distribution between the video and sensor results revealed the swing end event detection was centered near -0.04 s by the Wilshin et al. method. Due to the early termination of swing phase, (b) swing duration was underestimated and (c) stance duration was overestimated.
(TIF)

## References

[1] Shin JH, Greer B, Hakim CH, Zhou Z, Chung YC, Duan Y, et al Quantitative phenotyping of Duchenne muscular dystrophy dogs by comprehensive gait analysis and overnight activity monitoring. PLoS One. 2013;8(3): e59875 doi: 10.1371/journal.pone.0059875 23544107 10.1371/journal.pone.0059875 PMC3609742

[2] Rathinam C, Bateman A, Peirson J, Skinner J. Observational gait assessment tools in paediatrics—a systematic review. Gait Posture. 2014;40(2): 279–285. doi: 10.1016/j.gaitpost.2014.04.187 24798609 10.1016/j.gaitpost.2014.04.187

[3] Cofré Lizama LE, Khan F, Lee PV, Galea MP. The use of laboratory gait analysis for understanding gait deterioration in people with multiple sclerosis. Mult. Scler. J. 2016;22(14): 1768–1776. 10.1177/1352458516658137 27364324

[4] Thewlis D, Bishop C, Daniell N, Paul G. Next-generation low-cost motion capture systems can provide comparable spatial accuracy to high-end systems. J. Appl. Biomech. 2013;29(1): 112–117. 22813783 10.1123/jab.29.1.112

[5] Picerno P. 25 years of lower limb joint kinematics by using inertial and magnetic sensors: a review of methodological approaches. Gait Posture. 2017;51: 239–246. doi: 10.1016/j.gaitpost.2016.11.008 27833057 10.1016/j.gaitpost.2016.11.008

[6] Duan D. Duchenne muscular dystrophy gene therapy in the canine model. Hum. Gene. Ther. Clin. Dev. 2015;26(1): 57–69. doi: 10.1089/humc.2015.006 25710459 10.1089/humc.2015.006 PMC4442571

[7] Simmons AD, Carrier DR, Farmer CG, Gregersen CS. Lack of locomotor-cardiac coupling in trotting dogs. Am. J. Physiol. Regul. Integr. Comp. Physiol. 1997;273:R1352–R1360. 10.1152/ajpregu.1997.273.4.R1352 9362299

[8] Barthélémy I, Barrey E, Aguilar P, Uriarte A, Le Chevoir M, Thibaud JL, et al Longitudinal ambulatory measurements of gait abnormality in dystrophin-deficient dogs. BMC Musculoskelet. Disord. 2011;12: 75 doi: 10.1186/1471-2474-12-75 21489295 10.1186/1471-2474-12-75 PMC3103492

[9] Pillard P, Gibert S, Viguier E. 3D accelerometric assessment of the gait of dogs with cranial cruciate ligament rupture. Comput Methods Biomech Biomed Engin. 2012;15: 129–131. doi: 10.1080/10255842.2012.713654 23009452 10.1080/10255842.2012.713654

[10] Clark K, Caraguel C, Leahey L, Béraud R. Evaluation of a novel accelerometer for kinetic gait analysis in dogs. Can. J. Vet. Res. 2014;78(3): 226–232. 24982555 PMC4068415

[11] Dewhirst OP, Roskilly K, Hubel TY, Jordan NR, Golabek KA, McNutt JW, et al An exploratory clustering approach for extracting stride parameters from tracking collars on free-ranging wild animals. J. Exp. Biol. 2017;220(3): 341. 27811292 10.1242/jeb.146035

[12] Duerr F, Pauls A, Kawcak C, Haussler KK, Bertocci G, Moorman V, et al Evaluation of inertial measurement units as a novel method for kinematic gait evaluation in dogs. Vet. Comp. Orthop. Traumatol. 2016;29(6): 475–483. doi: 10.3415/VCOT-16-01-0012 27761576 10.3415/VCOT-16-01-0012

[13] Wilshin S, Reeve MA, Haynes GC, Revzen S, Koditschek DE, Spence AJ. Longitudinal quasi-static stability predicts changes in dog gait on rough terrain. J. Exp. Biol. 2017;220(10): 1864–74 28264903 10.1242/jeb.149112 PMC5450805

[14] Ladha C, O’Sullivan J, Belshaw Z, Asher L. Gaitkeeper: A system for measuring canine gait. Sensors. 2017;17(2): 309. 10.3390/s17020309 PMC5335924 28208707

[15] Rhodin M, Bergh A, Gustås P, Gómez Álvarez CB. Inertial sensor-based system for lameness detection in trotting dogs with induced lameness. Vet. J. 2017;222: 54–59. doi: 10.1016/j.tvjl.2017.02.004 28283369 10.1016/j.tvjl.2017.02.004

[16] Alexander RM. The gaits of bipedal and quadrupedal animals. Int. J. Robotics Res. 1984;3: 49–59.

[17] Biknevicius AR, Reilly SM. Correlation of symmetrical gaits and whole body mechanics: debunking myths in locomotor biodynamics. Exp. Zool A. Comp. Exp. Biol. 2006;305A(11): 923–934. 10.1002/jez.a.332 17029269

[18] Maes LD, Herbin M, Hackert R, Bels VL, Abourachid A. Steady locomotion in dogs: temporal and associated spatial coordination patterns and the effect of speed. J. Exp. Biol. 2007;211(1): 138. 10.1242/jeb.008243 18083742

[19] Kano WT, Rahal SC, Agostinho FS, Mesquita LR, Santos RR, Monteiro FOB, et al Kinetic and temporospatial gait parameters in a heterogeneous group of dogs. BMC Vet. Res. 2016;12(1): 2. 26728918 10.1186/s12917-015-0631-2 PMC5015230

[20] Marsh AP, Eggebeen JD, Kornegay JN, Markert CD, Childers MK. Kinematics of gait in Golden Retriever Muscular Dystrophy. Neuromuscul Disord. 2010;20:16–20. doi: 10.1016/j.nmd.2009.10.007 19932618 10.1016/j.nmd.2009.10.007 PMC3777817

[21] Gillette RL, Angle TC. Recent developments in canine locomotor analysis: a review. Vet J. 2008;178: 165–176. doi: 10.1016/j.tvjl.2008.01.009 18406641 10.1016/j.tvjl.2008.01.009

[22] Chen S, Lach J, Lo B, Yang GZ. Toward pervasive gait analysis with wearable sensors: a systematic review. IEEE J. Biomed. Health Inform. 2016;20: 1521–1537. doi: 10.1109/JBHI.2016.2608720 28113185 10.1109/JBHI.2016.2608720

[23] Hildebrand M. The quadrupedal gaits of vertebrates: the timing of leg movements relates to balance, body shape, agility, speed, and energy expenditure. BioScience. 1989;39: 766–775.

[24] Gorton GE, Hebert DA, Gannotti ME. Assessment of the kinematic variability among 12 motion analysis laboratories. Gait and Posture. 2009;29: 398–402. doi: 10.1016/j.gaitpost.2008.10.060 19056271 10.1016/j.gaitpost.2008.10.060

[25] Gustås P, Pettersson K, Honkavaara S, Lagerstedt AS, Byström A. Kinematic and spatiotemporal assessment of habituation to treadmill walking in Labrador retrievers. Acta Vet. Scand. 2016;58: 87 doi: 10.1186/s13028-016-0265-9 28031036 10.1186/s13028-016-0265-9 PMC5192580

