# Supplementary figures and images for: Automatic characterization of stride parameters in canines with a single wearable inertial sensor
Source: PLoS One. 2018 Jun 14;13(6):e0198893. doi: 10.1371/journal.pone.0198893 (PMC6002023; doi:10.1371/journal.pone.0198893)

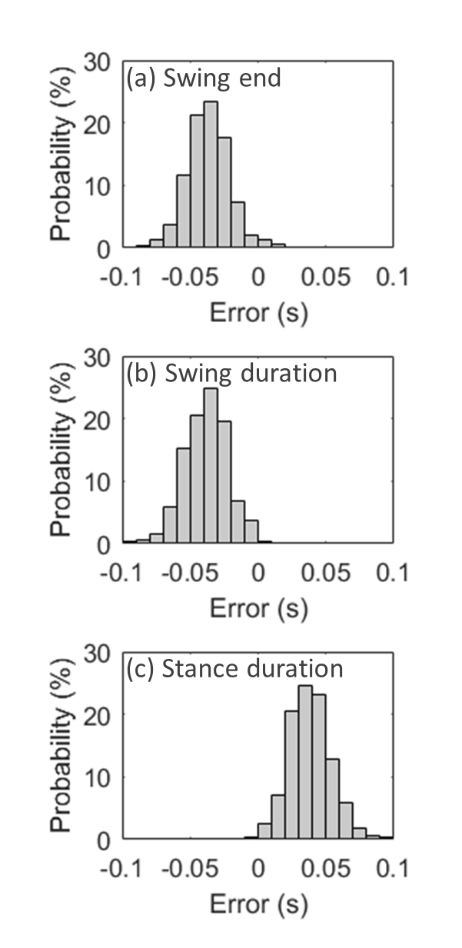

Supplement: S1 Fig — (a) Step-by-step error distribution between the video and sensor results revealed the swing end event detection was centered near -0.04 s by the Wilshin et al. method. Due to the early termination of swing phase, (b) swing duration was underestimated and (c) stance duration was overestimated. (TIF) [file pone.0198893.s002.tif]
